# Supplementary figures and images for: Transcriptional signatures in human macrophage-like cells infected by Leishmania infantum, Leishmania major and Leishmania tropica
Source: PLoS Negl Trop Dis. 2024 Apr 5;18(4):e0012085. doi: 10.1371/journal.pntd.0012085 (PMC11023634; doi:10.1371/journal.pntd.0012085)

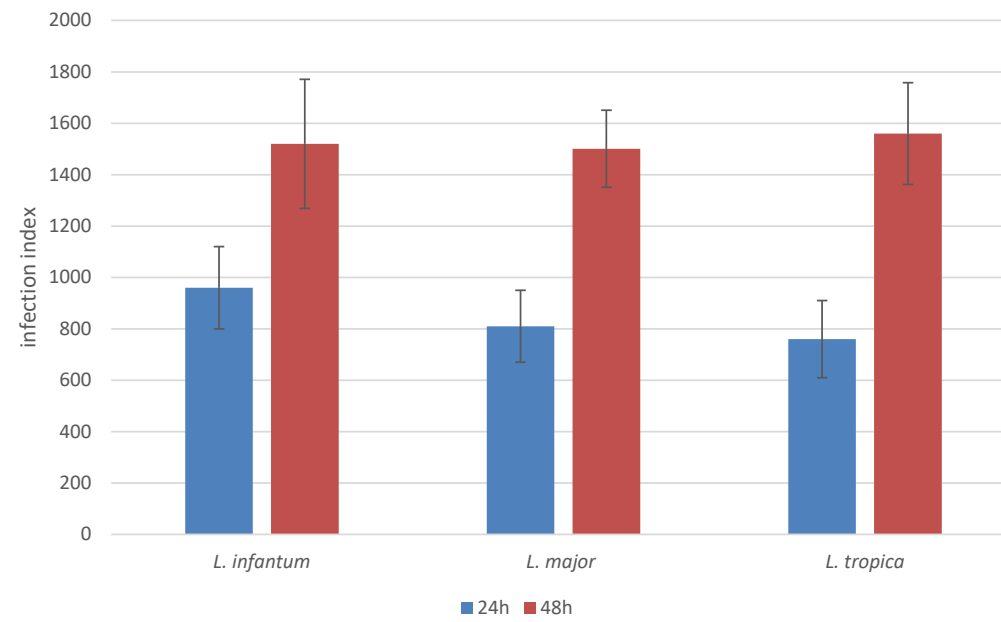

Supplement: S1 Fig — (PDF) [file pntd.0012085.s001.pdf]

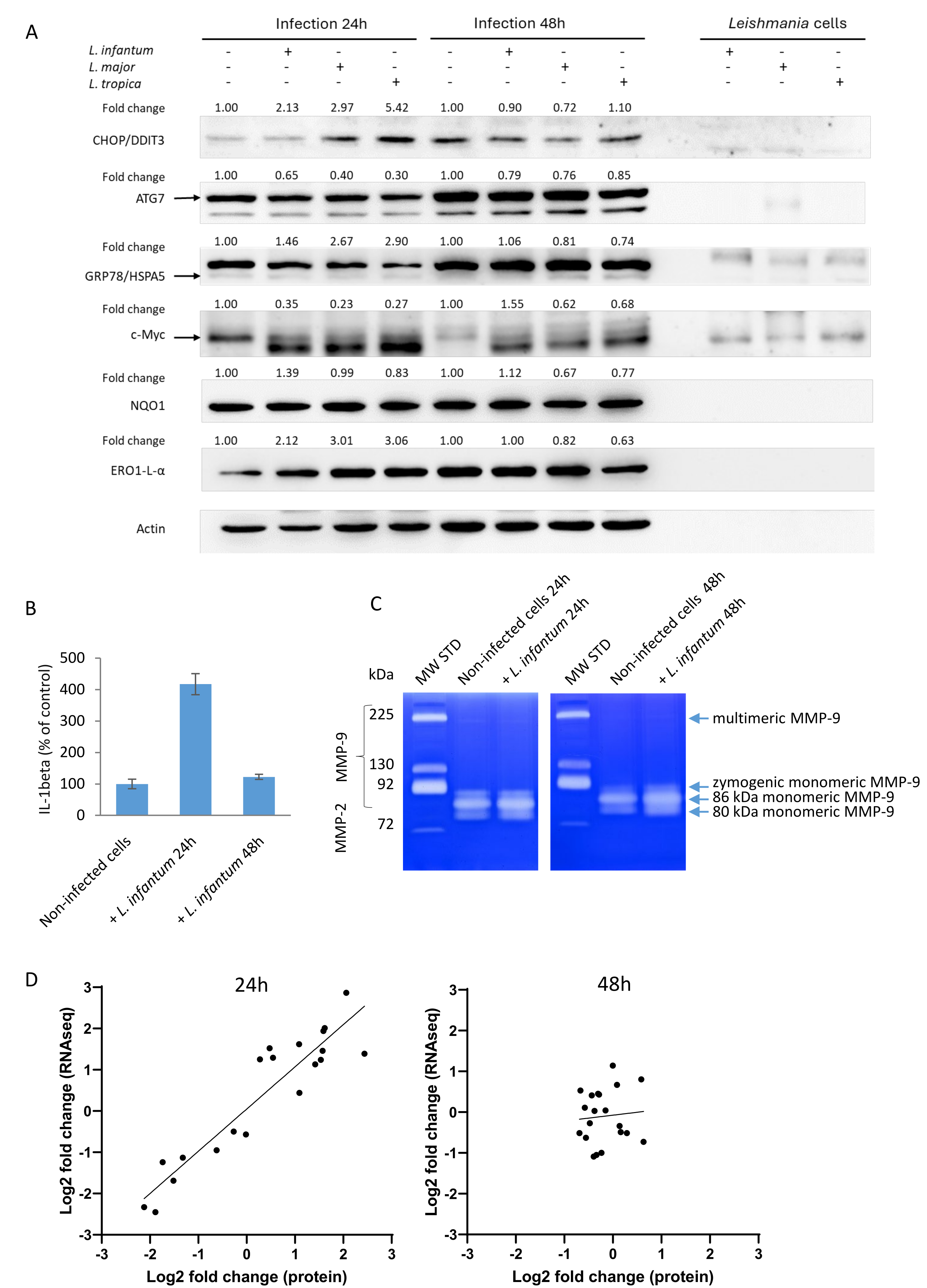

Supplement: S2 Fig — A) Representative western blot showing the evaluation of DDIT3, ATG7, ERO1-L-α (ERO1A), NQO1, HSP70 family protein 5 (HSPA5), and c-Myc (MYC) proteins in U937-derived macrophages infected with L. infantum, L. major, and L. tropica for 24h and 48h. Actin was used as loading control. The protein levels were analyzed in total cell lysates, and band density quantification was performed using a Chemi-Doc System. Densitometry values for specific proteins normalized against non-infected cells are included above each lane. B) IL-1β normalized to non-infected cells measured in supernatant of L. infantum-infected cells through ELISA test; C) Gelatin zymography of lysates from U937 cells are characterized by gelatinase’s forms belonging to the MMP-9 class; all gelatinolytic bands appear more evident in lysates from L. infantum-infected cells, in particular at 48h post-infection. D) Correlation analysis of Log2 fold change of mRNAs (determined by RNA-seq) and the corresponding proteins (determined by western blot, ELISA or gelatin zymography), at 24h post-infection (Spearman’s rho, ρ = 0.87, p<0.001), and 48h post-infection (Spearman’s rho, ρ = 0.08, p = 0.7). (TIF) [file pntd.0012085.s002.tif]
