# Supplementary material for: Transcriptional signatures in human macrophage-like cells infected by Leishmania infantum, Leishmania major and Leishmania tropica
Source: PLoS Negl Trop Dis. 2024 Apr 5;18(4):e0012085. doi: 10.1371/journal.pntd.0012085 (PMC11023634; doi:10.1371/journal.pntd.0012085)
Supplement: Attachment — Submitted filename: reviewers response final.docx [file pntd.0012085.s009.docx]

**Reviewer's Responses to Questions**

Key Review Criteria Required for Acceptance?
As you describe the new analyses required for acceptance, please consider the following:

**Methods**
-Are the objectives of the study clearly articulated with a clear testable hypothesis stated?
-Is the study design appropriate to address the stated objectives?
-Is the population clearly described and appropriate for the hypothesis being tested?
-Is the sample size sufficient to ensure adequate power to address the hypothesis being tested?
-Were correct statistical analysis used to support conclusions?
-Are there concerns about ethical or regulatory requirements being met?

**Reviewer #1**: See all relevant comments "Summary and General Comments" below.

**Reviewer #2:** The Methods are well-described and include all necessary information. One missing data need be included: the software(s) used to make data analysis and graph construction.

REPLY: softwares for data analysis were indicated in methods section. In particular STAR (with -quantMode GeneCounts) was used to map and count reads, while edgeR was used to perform the differential gene expression analysis [lines 145-147, original version]. Metascape was used to perform gene ontology analysis [line 156, original version].

We added information regarding graph construction [lines 155-156 revised version].

**Reviewer #3**: Are the objectives of the study clearly articulated with a clear testable hypothesis stated? Generally yes.
-Is the study design appropriate to address the stated objectives? In overall design, but a little short on execution.
-Is the population clearly described and appropriate for the hypothesis being tested? Generally yes, but see next point.
-Is the sample size sufficient to ensure adequate power to address the hypothesis being tested? Regarding inferences they were making regarding differences between the clinically distinct species, the sample size was insufficient (see general comments section).
-Were correct statistical analysis used to support conclusions? The number of clinically distinct species was very low, with two representatives from one and only one from the other. This would explain why there was no statistical analysis for this major aspect of their study

REPLY: This aspect has been pointed out in the revised manuscript (see also replies below)

-Are there concerns about ethical or regulatory requirements being met? No concerns.

--------------------

**Results**
-Does the analysis presented match the analysis plan?
-Are the results clearly and completely presented?
-Are the figures (Tables, Images) of sufficient quality for clarity?

**Reviewer #1**: See all relevant comments "Summary and General Comments" below.

**Reviewer #2**: The results and discussion are clear and well-presented in the text.

**Reviewer #3**: -Does the analysis presented match the analysis plan? Yes
-Are the results clearly and completely presented? Yes
-Are the figures (Tables, Images) of sufficient quality for clarity? Yes
--------------------

**Conclusions**
-Are the conclusions supported by the data presented?
-Are the limitations of analysis clearly described?
-Do the authors discuss how these data can be helpful to advance our understanding of the topic under study?
-Is public health relevance addressed?

**Reviewer #1**: See all relevant comments "Summary and General Comments" below.

**Reviewer #2**: The conclusions are supported by the data. Limitations could be including, mainly about the type of cell used for the work.

REPLY: see comments below

**Reviewer #3**: -Are the conclusions supported by the data presented? The conclusions are overly speculative at times.
-Are the limitations of analysis clearly described? No
-Do the authors discuss how these data can be helpful to advance our understanding of the topic under study? Somewhat.
-Is public health relevance addressed? Yes

REPLY: see comments below

--------------------

**Editorial and Data Presentation Modifications?**
Use  this section for editorial suggestions as well as relatively minor modifications of existing data that would enhance clarity. If the only modifications needed are minor and/or editorial, you may wish to recommend “Minor Revision” or “Accept”.

**Reviewer #1**: See all relevant comments "Summary and General Comments" below.

**Reviewer #2**: I have only fewer comments.
•  In line 234, use: genus Leishmania OR subgenus Leishmania (Leishmania);

REPLY: modified using subgenus Leishmania (Leishmania)

• Figure 3: include the terms Upregulated and Downregulated above diagram columns (like 24h and 48h titles for lines)

REPLY: DONE

• To make clearer, authors could carefully revise the gene and protein nomenclature, some terms (as in lines 300 and 317) are referring to proteins but graphed without hyphen.

REPLY: the gene and protein nomenclature has been revised

• Line 421: use BALB/c

REPLY: DONE

•       The colors used in the Figure 4 are quite confusing. Could you change to two colors pattern (one for downregulated and other to upregulated genes)?

REPLY: Unfortunately, it was not possible to change colors in figure 4 (which was generated in Metascape); however, panel A is referred to upregulated genes while panel B to downregulated genes. The color shade reflects the significance [i.e., -log10(p value)] of perturbed pathway in the different species, as indicated in the top-right legends, with the darkest representing the most significant.
 **Reviewer #3**: (No Response)

--------------------

**Summary and General Comments**

**Reviewer #1:** In this manuscript, the authors carried out transcriptomic analyses on human macrophage-like cells (PMA-differentiated U937) infected with three Leishmania species, namely L. infantum, L. major, and L. tropica. Transcriptional signatures analyses revealed common but also differential host gene expression trends between the different parasite species. Furthermore, host gene expression was assessed at two different time points, offering a better appreciation of the kinetics of gene expression. This study adds incremental knowledge of the transcriptional perturbations caused by Leishmania parasites. Overall, the study design is straightforward, and analyses appear to be appropriately carried out. A few points should be addressed by the authors before accepting the manuscript for publication.

1.  Although the use of PMA to differentiate monocytic-like cell lines towards macrophage-like cells (ex: THP-1, U937) is standard practice, it is well recognized that PMA also transiently activates cells to produce inflammatory mediators (amongst other things). It would therefore be pertinent to mention this point in the discussion as it is undoubtedly a variable that influences gene expression and hence interpretation of the results.

REPLY: We agree with referee, however the control cells to calculate gene expression were equally differentiated with PMA but non infected, therefore the effect of PMA is subtracted. This has been better specified in methods [line 121]

2.  In the Methods section, the authors indicate that “Infections were repeated twice (one dish per infection)” (see line 120). Could the author clarify whether the infections were performed in two independent biological experiments, or if the replicates correspond to technical replicates (i.e., two separate plates but prepared as part of a single experiment)?

REPLY: The infections were performed in two independent experiments. This has been better pointed out [lines 121-122].

3.  In the Results and Discussion section, the authors state that “[…] the trimmed reads mapped on the human were 44.6% and 66.1% at 24 and 48 h, respectively, while for the uninfected macrophages were 98.5% and 98.8% at 24 h and 48 h, respectively” (see lines 189-191). This raises a few questions:

•  Were the mapped reads from samples infected from the different Leishmania species similar? It is not clear to which samples these values (i.e., 44.6% and 66.1%) correspond. Can the authors clarify?

REPLY: The values 44.6% and 66.1% were average for all infected species at 24h and 48h post infection, respectively. This has been better specified. Moreover, to give an indication of consistency, the standard error has been added [lines 232-234].

•  The values of mapped reads obtained from infected samples seem somewhat low, especially compared to uninfected samples. Presumably, this is due to “contaminating” reads coming from parasite-derived transcripts. Have the authors considered first filtering out reads that would map to the Leishmania genome prior to performing their sequence alignments on the human genome? In any case, can the authors elaborate on this point?

REPLY: We agree that this is due to reads coming from parasite transcripts. However, since the two genomes (human and Leishmania) are so different and considering the stringency of STAR during the mapping steps, we did not perform any filtering of leishmania reads before to map reads on human genome. The fact that the reduction of mapped reads from infected samples was due to parasite-derived transcripts was confirmed by aligning reads to Leishmania genomes: in these cases, the sum of percentages of reads aligned to human genome and Leishmania genome in the infected samples was close to the percentage of reads aligned to human genome in the uninfected controls. This fact has been mentioned in the text [lines 234-237]

4.  Although RT-qPCR analyses agree to a high degree and validate the transcriptomic analyses, the study would greatly benefit from the inclusion of a handful of validation at the protein level. Indeed, this would help show potential functional changes in the infected host cell, rather than simply at a mRNA level (which do not always correlate with biologically meaningful changes in protein expression and thus function). Have the authors monitor any of the regulated genes at the protein level?
REPLY: We agree with referee. To fulfill this request, we evaluated the expression of several proteins [DDIT3, ATG7, ERO1-L-alpha (ERO1A), NQO1, HSP70 family protein 5 (HSPA5), c-Myc, IL-1b, MMP-9] in control and infected cells through western blot, ELISA test or gelatin zymography. Consequently, Materials and methods and Results/discussion sections have now been implemented [lines 182-219; lines 271-279] and a supplementary figure has been added (S2 Fig)

**Reviewer #2**:

Dear authors,
Firstly, congrats for the manuscript. It is a very interesting work and well-conduced. The manuscript writing has higher quality. The only missing part: I agree with you, the importance of make analysis in human macrophages, but these used cells are a lineage originated from cancer and differentiated using inflammatory activator. Please, you need clarify that these data could be a limitation of obtained results.
REPLY: We thank the referee for the positive comments. We also agree with the limitation highlighted by the referee. This aspect has been mentioned in the revised manuscript [lines 360-361; lines 597-600]

**Reviewer #3**: The distinct clinical manifestations of Leishmaniasis are largely associated with different Leishmania species. L. major and L. tropica, for example, are typically associated with cutaneous leishmaniasis, whereas species including L. infantum are usually associated with visceral leishmaniasis. The factors that determine these species-specific clinical differences are poorly understood. To address this medically important gap in knowledge, the authors examine macrophage transcriptional responses to Leishmania both common amongst, and distinct between these three Leishmania species.

Strengths:
The authors have identified macrophage transcriptional signatures common to infection with all three of these Leishmania species and also signatures specific to the two cutaneous species versus the visceral species.
Although comparing macrophage gene expression upon infection with a cutaneous vs. a visceralizing species is not entirely novel, this major aspect of their study nonetheless represents a new contribution to this important, but neglected area of research.

Concerns:
These in vitro studies of the immortalized monocyte-like U937 line have somewhat limited physiologic relevance. Further, this study only focuses on the macrophage part of the response. It does not include other innate cells nor the adaptive response and other tissue specific aspects involved in the vivo response. The authors should mention these limitations.
REPLY: These limitations have now been mentioned in the Results/discussion [lines 360-361] and conclusions [lines 597-600] (See also reply to reviewer 2).

It is unclear why so few transcripts were validated by RT-qPCR (apparently they only validated only 8 at the 24 hr timepoint). Instead of focusing on the few genes that were validated, the discussion of species-specific genes/pathways was focused on genes whose expression was not validated. This is a major disconnect that needs to be addressed experimentally, at their 24 and 48 hr timepoints

REPLY: In the original version of the manuscript, 8 transcripts were validated at both 24h and 48h post-infection. In the revised version, additional transcripts connected to discussed pathways have been validated. As a whole, the expression of 18 dysregulated genes has been verified by qPCR in cells infected by L. infantum, L. major and L. tropica, either at 24h and 48h post infection (i.e., 108 total assays) [lines 266-267]. The results showed a general good correlation between qPCR and RNA-seq for all infecting species, further strengthening the validation of RNA-seq results. The figure 2 and its legend has been modified according to new data.

The authors emphasize that their study represents a systematic comparison of macrophages infected with Leishmania species that cause different disease outcomes, however to really be systematic and statistically valid, the work should include more representative species from each disease presentation. For example, why not at least include L. donovani, a visceralizing species that is also present in the Mediterranean, as well? Further, have the authors considered analyzing/discussing dermotropic strains of  L. infantum that are also circulating in the Mediterranean region?

REPLY: in mediterranean region the most represented viscerotropic species is L. infantum, even if L. donovani has been found in eastern Mediterranean (Cyprus). In our work we used the L, infantum reference strain MHOM/IT/08/31U. The use of dermotropic L. infantum strains will be certainly useful to study the pathogen or host determinants for visceralization. We thank the referee for his suggestion that we will try to implement in a future work. This aspect has been added in the results/discussion section [lines 565-568].

It would be more informative if the authors included a brief discussion of how/why some of their major findings may have differed from other studies of Leishmania species-specific effects on macrophage gene expression (e.g. Cox2).

REPLY: Many findings were confirmed in other studies, as indicated in the original version of manuscript (see pages 14, 16, 22, 23). Concerning Cox2/PTGS2, we found it significantly upregulated at 24h in cells infected by L. infantum, confirming data obtained in macrophages infected with the closely related species L. donovani (Bhattacharjee A et al Infect Immun. 2016;84:2963–73) (lines 307-309 original manuscript). Nevertheless, some findings regarding pathways could be different from other studies due to several reasons: i) different models (i.e., host cell and Leishmania species or strain), ii) time points, iii) infection rates, iv) bioinformatics tools used and v) information available for data mining analysis (GO database, reactome, etc), which are growing fast.

Overall, the study is somewhat unfocused. Much of the time, they are just listing individual genes they identified, with standard descriptions of their reported functions. Although potentially interesting, without a functional follow-up, this does not contribute much insight into the determinants of clinically distinct forms of leishmaniasis. The authors might be better served keeping many of these genes in their supplementary data and instead focus their descriptions/discussion on the RT-qPCR-validated genes.

REPLY the qPCR validated genes have been increased adding genes involved in pathways that have been discussed. In any case, the qPCR results were aimed to validate the entire set of dysregulated genes found by RNA seq. Therefore, the discussion was focused on pathways significantly affected globally. However, new data on several genes involved in the pathways discussed have been added (see reply below in summary section).

Minor points:
They used stationary phase promastigotes, ideally would be better to use purified metacyclics.

REPLY: We agree with the referee, but also in this case the infection was well established as demonstrated by infection index (S1 fig).

A phagocytosis control, such as latex beads as others have used, would have been more informative.

REPLY: This condition is effectively lacking; however, the main goal was to find differences between dermotropic or viscerotropic species, and the effect of phagocytosis should be the same for all species.

Line 333: “CCL23 was newly downregulated”; unclear what the authors mean here. Why not just say “down regulated”, as with the other 24hr p.i. genes, or is there something temporally different about CCL23?
REPLY: In this case the gene was downregulated only at 48h (not at 24h). This has been better explained (lines 390-391)

Many minor issues with use of language, grammar and a few typos:
e.g.: Line 20: “Despite animal models and genomic/transcriptomic studies provided important insights, the pathogenic determinants….are still poorly understood”

REPLY: MODIFIED

e.g. : Line 2-309: “ infected cells respect to” (should read “ infected cells with respect to”)

REPLY: MODIFIED

e.g. Lines 279-280 sound a bit odd: contrary to the authors assertions, genes don’t “unravel pathways”. MODIFIED with “provide information about” [line 336]

e.g. Line 381: what is a “multiple list”? the term multiple list is referred to a table containing three lists of deregulated genes, one for each infecting species, which have been submitted to metascape for meta analysis. This has been better explained in the method section [lines 159-160].

Summary
Overall, the authors should make clear that, whilst interesting and representing a contribution to knowledge in an important area, their findings represent a rather limited dataset (i.e. they present an in vitro study of the response of a single immortalized monocytic-like cell line to several Leishmania species, with a sample size insufficient to provide a meaningful stand-alone comparison between cutaneous and visceralizing Leishmania species). Importantly, the genes/pathways highlighted in their principal findings do not appear to have been validated. It is important that they validate these genes.

REPLY. The number of strains used has been mentioned and commented in the manuscript (see also replies above). The number of genes validated by qPCR and/or at the protein level has been considerably increased. Some of these genes are part of pathways that have been discussed: e.g., VEGFA-VEGFR2 signaling pathway (MMP10, JUN, STAT1); IL17 signaling (IL1B, IL6, MMP3, MMP9, JUN); NFE2L2 regulating anti-oxidant/detoxification enzymes (NQO1, HMOX1); unfolded protein response (DDIT3, HSPA5); interferon signaling (IFI6, IFIT1, OAS2, STAT1); cholesterol and lipid metabolism (DHCR7, ACAT2, HMGCR, HMGCS1); MHC class II antigen presentation (MYC), neutrophil degranulation (ATG7), etc.

The authors should avoid over-interpretation/extrapolation.  For example, one of their principal, yet unvalidated findings was that genes associated with the VEGF pathway were upregulated in L. infantum-infected cells. They interpreted this to indicate that vascular remodeling may be important for visceralization. Aside from the apparent lack of validation for VEGF genes, this interpretation remains overly speculative, especially since VEGF is known to also have roles outside of the endothelial/vascular system.
REPLY: The VEGF gene was not significantly upregulated *per se* (p=0.08). However, please consider that the entire pathway (which included many dysregulated genes) was significantly affected. Moreover, some dysregulated gene involved in VEGFA-VEGFR2 signaling pathway (e.g., MMP10, JUN, STAT1) (see table S5) have been validated also by qPCR. However, our findings are limited to our infection model, as pointed out in the revised manuscript (see also replies above).
--------------------
